# Management of malignant pleural mesothelioma – part 3: Data from the Austrian Mesothelioma Interest Group (AMIG) database

**DOI:** 10.1007/s00508-016-1037-2

**Published:** 2016-07-25

**Authors:** Thomas Klikovits, Mir Alireza Hoda, Yawen Dong, Madeleine Arns, Bernhard Baumgartner, Peter Errhalt, Christian Geltner, Barbara Machan, Wolfgang Pohl, Jörg Hutter, Josef Eckmayr, Michael Studnicka, Martin Flicker, Peter Cerkl, Klaus Kirchbacher, Walter Klepetko

**Affiliations:** 1 Division of Thoracic Surgery, Department of Surgery, Comprehensive Cancer Center, Medical University Vienna, Waehringer Guertel 18–20, 1090 Vienna, Austria; 2 Department of Pulmonology, LKH Hochegg, Hochegg, Austria; 3 Department of Pulmonology, Landeskrankenhaus Vöklabruck, Vöklabruck, Austria; 4 Department of Pulmonology, University Clinic Krems, Krems, Austria; 5 Department of Pulmonology, Klinikum Klagenfurt, Klagenfurt, Austria; 6 Rehabilitation Center Tobelbad, Allgemeine Unfallversicherungsanstalt, Tobelbad, Austria; 7 Department of Pneumology, KH Hietzing, Karl Landsteiner Institute for Clinical and Experimental Pneumology, Vienna, Austria; 8 Department of Surgery, University Clinic Salzburg, Salzburg, Austria; 9 Department of Pulmonology, Landeskrankenhaus Wels, Wels, Austria; 10 Department of Pulmonology, University Clinic Salzburg, Salzburg, Austria; 11 Department of Pulmonology, Landeskrankenhaus Leoben, Leoben, Austria; 12 Department of Pulmonology, Landeskrankenhaus Hohenems, Hohenems, Austria; 13 Department of Pulmonology, Wilhelminenspital Vienna, Vienna, Austria

**Keywords:** Malignant pleural mesothelioma, Tumor registry, Epidemiology, Treatment, Outcome

## Abstract

**Background:**

Malignant pleural mesothelioma (MPM) is a rare but aggressive tumor originating from the pleural cavity with a strong link to previous asbestos exposure. In order to determine the demographics, diagnostics, therapeutic strategies, and prognosis of MPM patients in Austria, the Austrian Mesothelioma Interest Group (AMIG) was founded in 2011. In this report the data from the AMIG MPM database collected to date are reported.

**Methods:**

A prospective observational registry was initiated, including patients with histologically verified MPM diagnosed and treated at specialized centers in Austria. Patient inclusion started in January 2011 and follow-up was completed until September 2015.

**Results:**

A total number of 210 patients were included. There were 167 male and 43 female patients with a mean age of 67.0 years (SD ± 11.3) at the time of diagnosis. Asbestos exposure was confirmed in 109 (69.4 %) patients. The histological subtype was epithelioid in 141 (67.2 %), sarcomatoid in 16 (7.6 %), biphasic in 28 (13.3 %), and MPM not otherwise specified in 25 (11.9 %) patients. Of the patients, 30 (14.3 %) received best supportive care (BSC) only, 71 (33.8 %) chemotherapy (CHT) alone, four (1.9 %) radiotherapy (RT) alone, 23 (11.9 %) CHT/RT, two (0.9 %) surgery alone, and 76 (36.2 %) curative surgery within a multimodality treatment (MMT), which was more frequently performed for patients younger than 65 years and with early-stage disease (I + II). Median overall survival (OS) was 19.1 months (95 % CI 14.7–23.5). The 1‑, 3‑, and 5‑year OS rates were 66 %, 30 %, and 23 %, respectively, and OS was significantly better in patients undergoing surgery within MMT (5-year survival 5 % vs. 40 %, *p* = 0.001).

**Conclusion:**

Patients with earlier disease stages, younger age, good performance status, and epithelioid histology were more likely to undergo MMT including surgery, which resulted in a more favorable outcome.

## Introduction

Mesothelioma is a rare malignant tumor arising from mesothelial cells lining the pleural, pericardial, or peritoneal cavity. The most common and most aggressive type is malignant pleural mesothelioma (MPM), which is strongly associated with previous asbestos exposure. The incidence of MPM is between 1 and 2 in 100,000 and is still rising in most European countries [1]. Clinical and pathological verification is challenging and the majority of patients have advanced-stage disease at the time of the first diagnosis [2]. The prognosis of the disease is dismal, with a median overall survival (OS) from 9 to 12 months [3]. Recently, the therapeutic management in early stages has shifted from single-therapy approaches to multimodality treatment strategies including chemotherapy (CHT), radiotherapy (RT), and radical surgery [4, 5]. Nevertheless, local spread and extensive tumor growth often prevent complete surgical removal. Thus, the high mortality and early progression of MPM is largely due to locoregional spread/recurrence within the pleural space or transdiaphragmatically into the abdominal cavity. Moreover, in advanced stages, symptom control and palliative attempts are needed for improved quality of life [6]. In order to determine the demographics, diagnostics, therapeutic strategies, and prognosis of patients with MPM, the Austrian Mesothelioma Interest Group (AMIG) was founded in 2011 involving all main centers treating these patients in Austria. Detailed information about the group can be obtained on the Internet (http://www.amig.at). Several meetings were held and the members of the AMIG decided to initiate a registry in order to collect and evaluate data on epidemiology, treatment, and outcome of MPM patients in Austria. These data were collected and analyzed from the AMIG MPM database and are reported in this manuscript.

## Patients and methods

**Fig. 1:**
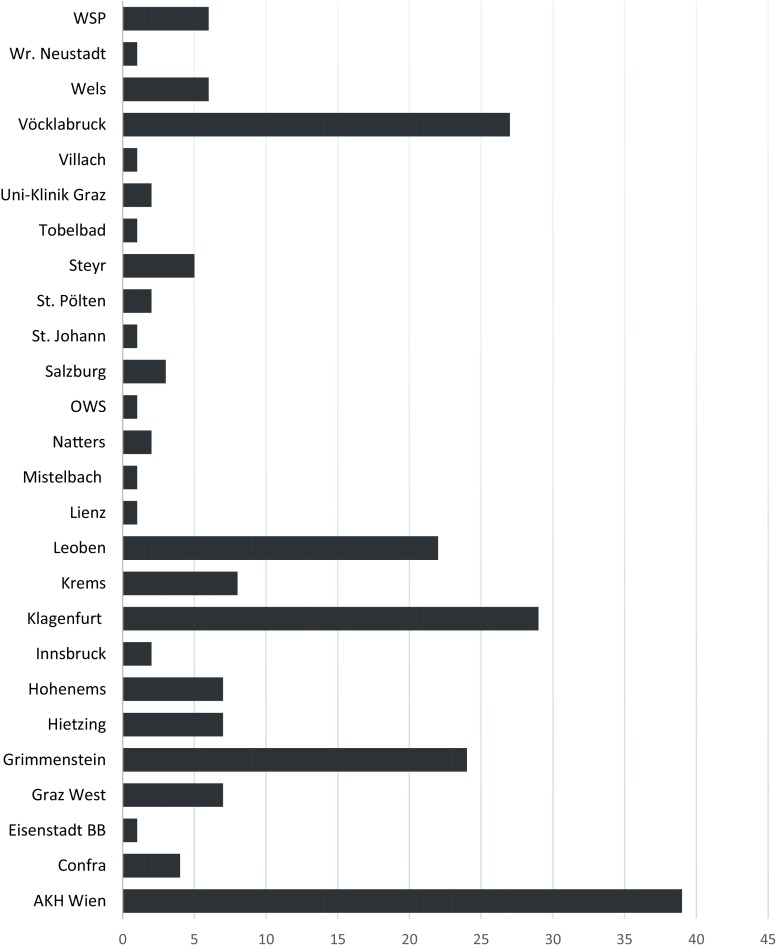
Number of patients included by the different centers in Austria

A prospective multi-institutional tumor registry was initiated involving 25 different centers in Austria (Fig. 1). Patients were included if MPM was histologically verified between January 2011 and July 2015 regardless of stage and treatment modality. Customized case report forms (CRF) were established and contributing centers were required to transfer CRFs to the AMIG coordination office at the Division of Thoracic Surgery, Medical University of Vienna at different time points: (1) at the time of diagnosis of MPM, (2) at the beginning of treatment, (3) at the end of treatment, and (4) at the last follow-up or until death. Patients were diagnosed, staged, and treated according to the common practice of the individual institution. The latest International Mesothelioma Interest Group (IMIG) staging system was routinely applied [2]. Follow-up was completed until September 2015. This prospective tumor registry was approved by the ethics committee of the Medical University of Vienna, serving as the leading ethics committee for this multi-institutional study. All procedures followed were in accordance with the ethical standards of the responsible committee on human experimentation (institutional and national) and with the Helsinki Declaration of 1975, as revised in 2008. Individual patient consent was required and only patients who gave written consent were included. CRFs were transferred to a digital database and data were anonymously saved at the AMIG coordination office. Interim analyses were presented at annual meetings of the participating AMIG members.

### Statistical analysis

Metric data are given as median and corresponding range, or, in the case of survival, as median and corresponding 95 % confidence interval (CI) if not otherwise indicated. OS was defined as the time between MPM diagnosis and death or, in censored patients, diagnosis and last follow-up date. Survival was analyzed by the Kaplan–Meier method and log rank test or by the Cox regression model to calculate hazard ratios (HRs) and corresponding CIs. The χ^2^ test was performed for analyzing the association between categorical factors. The correlation of metric data was analyzed by Pearson’s correlation coefficient. The threshold of significance was set at *p* < 0.05; *p* values are given as two-sided. The SPSS V. 20 software package (IBM, Armonk, N.Y.) was used for all statistical analyses.

## Results

### Patient demographics

In total, 210 patients with histologically verified MPM were included. There were 167 (79.5 %) male and 43 (20.5 %) female patients with a mean age of 67.0 years (SD ± 11.3) at the time of diagnosis. Asbestos exposure was confirmed in 109 (69.4 %) patients. Smoking history was known in 166 (79 %) patients, of which 72 (43.4 %) were never smokers, 73 (44 %) former smokers, and 21 (12.6 %) current smokers. The mean Karnofsky index at the time of diagnosis was 84.85 (SD ± 11.8). The histological subtype was epithelioid in 141 (67.2 %), sarcomatoid in 16 (7.6 %), biphasic in 28 (13.3 %), and MPM not otherwise specified in 25 (11.9 %) patients. Among the main initial symptoms, dyspnea of unclear origin (62.8 %), recurrent or persisting pleural effusion (41 %), and thoracic pain (29 %) were most frequent (Table 1).

**Table 1:** Basic data of 210 patients with MPM documented in the AMIG MPM registry

	Number (*n*)	%
Gender (male), *n* = 210	167	79.5
Mean age, *n* = 210	67 years (±11.3)	
Mean Karnofsky index, *n* = 168	85	
Mean follow-up	468.4 days	
Asbestos exposure, *n* = 157	109	69.4
Histology, *n* = 210		
Epithelioid Biphasic Sarcomatoid Not specified	141 28 16 25	67.2 13.3 7.6 11.9
IMIG staging , *n* = 150		
I II III IV	26 23 40 61	17.3 15.3 26.7 40.7
Staging modality, *n* = 198		
CT PET PET-CT	198 21 128	100 10.6 64.6
Diagnostic modality, *n* = 210		
Pleural biopsy Pleural puncture Others	164 18 28	78.1 8.6 13.3

*AMIG* Austrian Mesothelioma Interest Group, *MPM* malignant pleural mesothelioma, *IMIG* International Mesothelioma Interest Group, *CT* computed tomography, *PET* positron emission tomography

### Diagnostic and staging procedures

Information on diagnostic and staging modalities was available for 198 (94.3 %) patients. In 164 (78.1 %) patients, a direct pleural biopsy was performed for histological verification. Of these, 89 (53.3 %) underwent diagnostic video-assisted thoracoscopic biopsy. In 18 (8.6 %) patients, pleural puncture was successful in establishing the diagnosis of MPM. In 198 (100 %) patients, computed tomography (CT) of the chest was performed, 128 (64.6 %) received additional combined positron emission tomography (PET)/CT, and 21 (10.6 %) a PET. Additional bronchoscopy was performed on 25 (11.9 %) patients. Data on initial staging were available for 150 (71.4 %) patients. Among these, stages were distributed as follows: I, *n* = 26 (17.3 %); II, *n* = 23 (15.3 %); III, *n* = 40 (26.7 %); IV, *n* = 61 (40.7 %) (Table 1).

### Treatment modalities and follow-up

In all, 30 (14.3 %) patients received best supportive care (BSC) only, 71 (33.8 %) chemotherapy (CHT) alone, four (1.9 %) radiotherapy (RT) alone, 23 (11.9 %) CHT/RT, two (0.9 %) surgery alone, and 76 (36.2 %) curative surgery within a multimodality treatment (MMT = surgery combined with either CHT, RT, or CHT/RT). For four patients (1.9 %), no data on treatment were available. The most common type of chemotherapy was a combination of cisplatin/pemetrexed (*n* = 100), and various drugs and combinations were used according to the treating oncologist. Surgical procedures consisted of 50 (23.8 %) extrapleural pneumonectomies (EPP), 19 (9 %) pleurectomy/decortications (P/D), and nine (4.3 %) other operations (e.g., partial pleurectomy en bloc with adjacent lung tissue) with the intent of macroscopic complete resection. In patients undergoing curative intent surgery, the postoperative complication rate was 21.8 % (*n* = 17) and 30-day mortality was nil. Re-evaluation and follow-up were routinely performed in 3‑ to 6‑month intervals with chest x‑ray and chest CT scan if necessary. At the end of the follow-up, 99 (47.1 %) patients had died. Of these patients, the cause of death was tumor related in 85 (85.9 %) cases.

### Survival

**Fig. 2:**
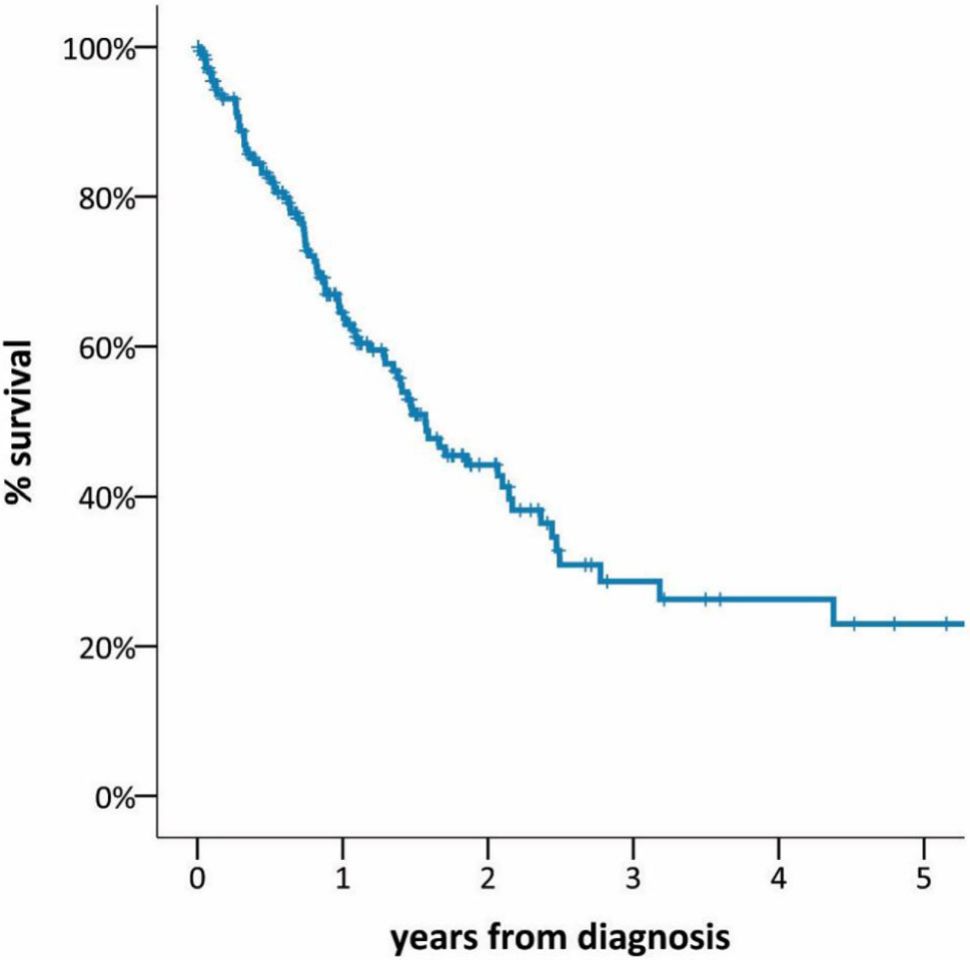
Overall survival of 185 patients with MPM

**Fig. 3:**
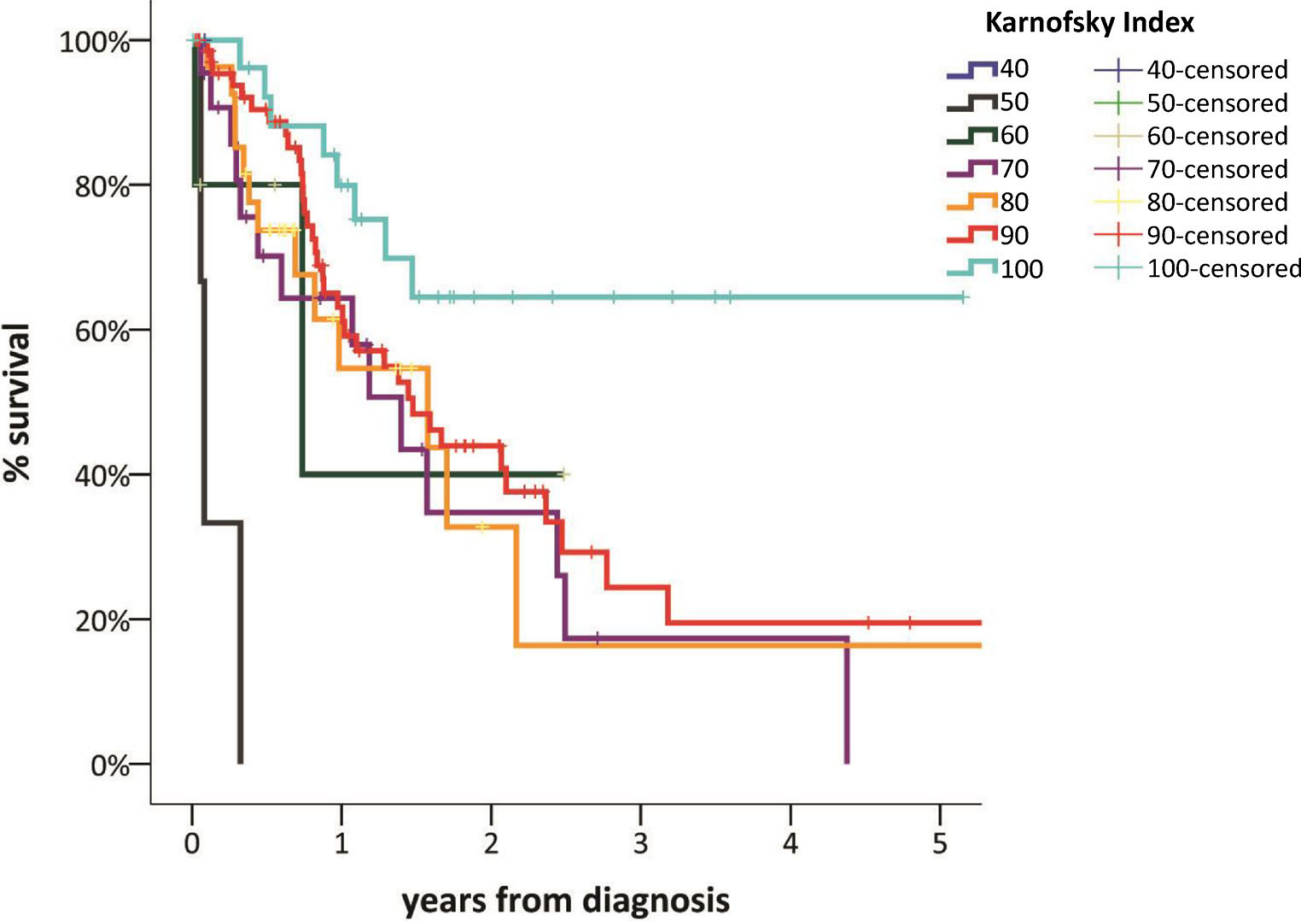
Survival according to the Karnofsky index regardless of histology, stage, and treatment

**Fig. 4:**
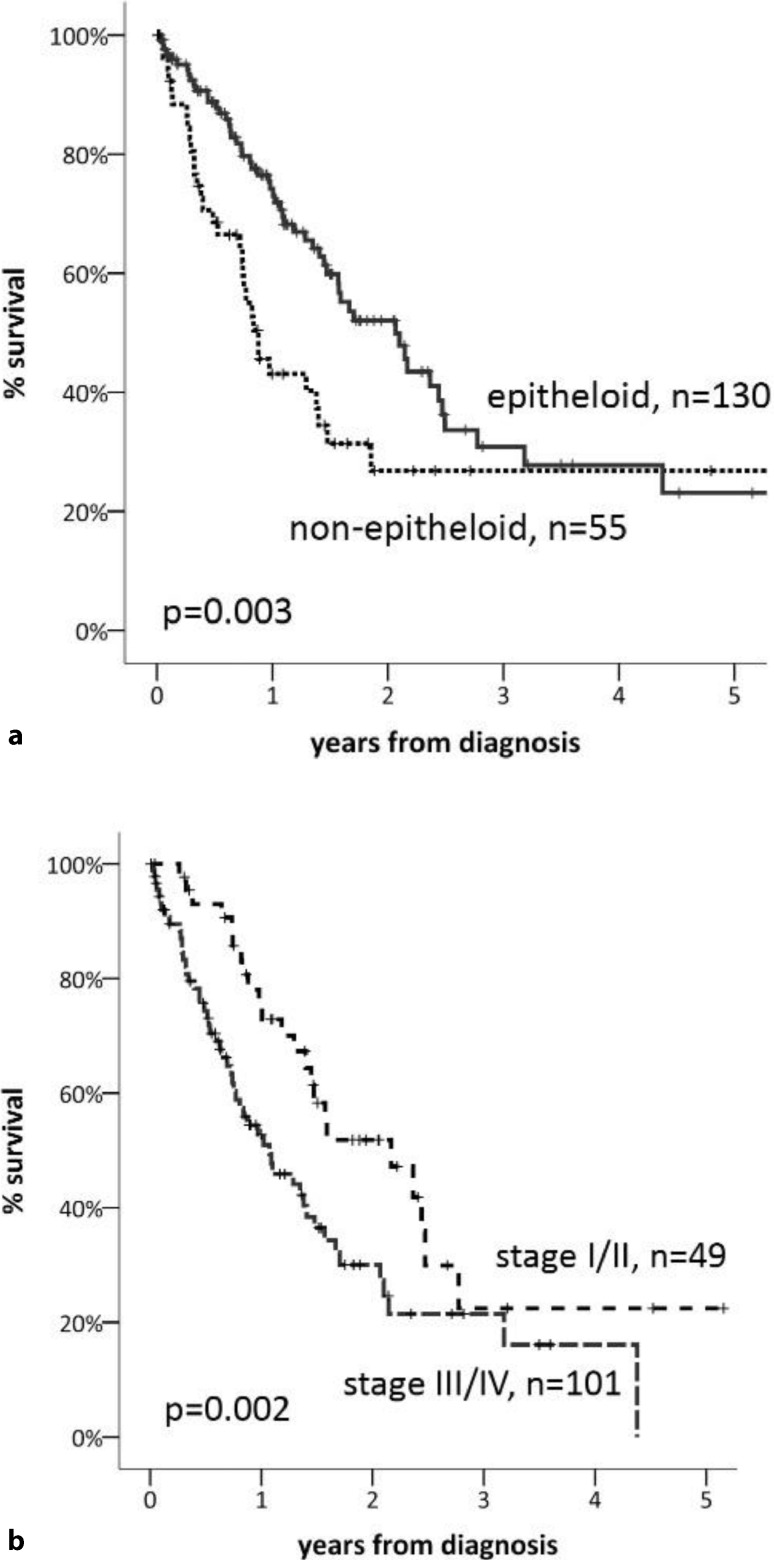
Survival in patients with malignant pleural mesothelioma according to histology (**a**) and stage (**b**)

**Fig. 5:**
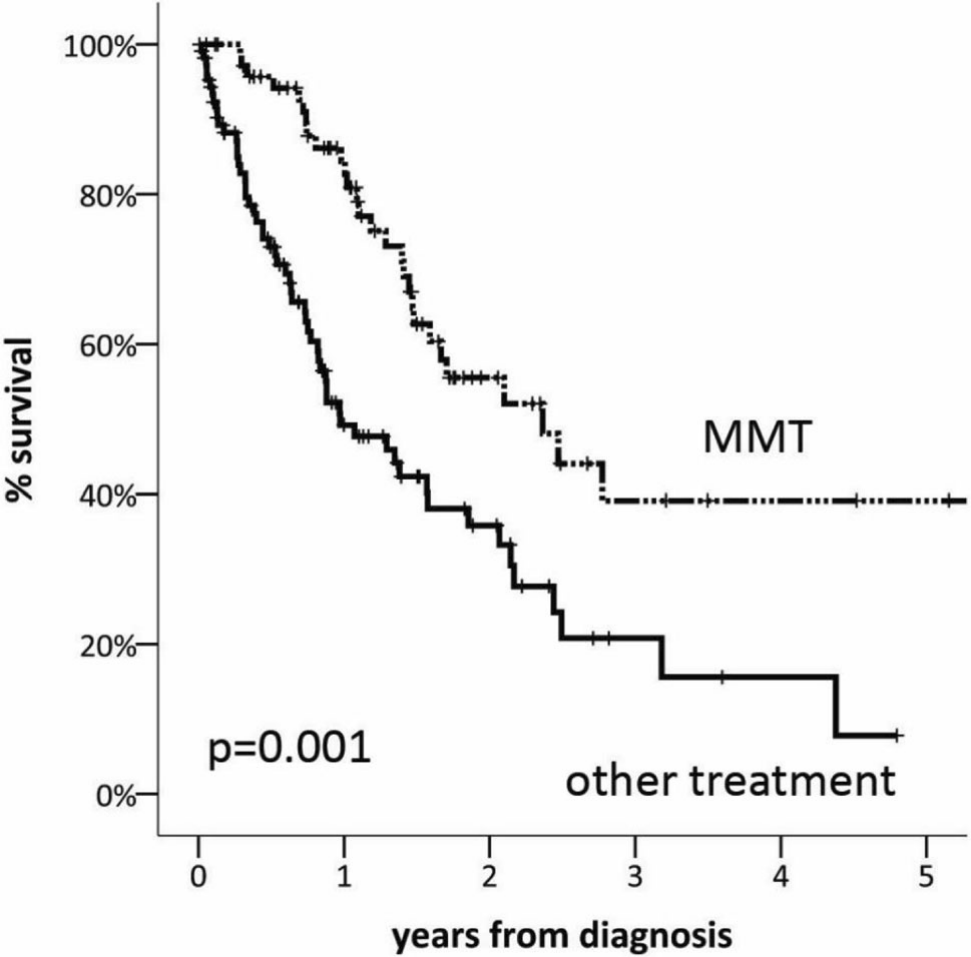
Survival of patients undergoing surgery within multimodality treatment (*MMT*) compared with patients with other treatment modalities

Sufficient data for survival analysis were available in 185 (88.1 %) patients. Median OS was 19.1 months (95 % CI 14.7–23.5). The 1‑, 3‑, and 5‑year OS was 66 %, 30 %, and 23 %, respectively (Fig. 2). When Kaplan–Meier survival was calculated for performance status measured by the Karnofsky index at the time of diagnosis, survival curves showed appropriate discrimination (Fig. 3). The survival analysis based on the IMIG staging system revealed that MPM patients with early-stage disease (I, II) had a significantly better outcome than those with late-stage disease (III, IV; median OS 26.4 vs. 13.0 months, HR 0.55, 95 % CI 0.35–0.86, *p* = 0.002). Patients with epithelioid histological subtype had improved OS compared with those with non-epithelioid subtype (median OS 25.1 vs. 10.2 months, *p* = 0.003; Fig. 4). There was no survival difference between males and females and patients below or above the age of 65 years. Survival was significantly improved in patients undergoing surgery within multimodality treatment compared with patients with other treatment modalities (5-year survival 40 % vs. 5 %, *p* = 0.001; Fig. 5). However, patients within MMT were significantly younger (age <65a, *p* = 0.001), had earlier-stage disease (stage I/II vs. III/IV, *p* = 0.001), better performance status (mean Karnofsky index 87.6 vs. 83.2, *p* = 0.017), and more often had epithelioid subtype (*p* = 0.001; Table 2). When performing a multivariate Cox regression survival analysis, performance status (HR 0.96, 95 % CI 0.94–0.96, *p* = 0.001) and histological subtype (non-epithelioid vs. epithelioid, HR 1.8, 95 % CI 1.06–3.07, *p* = 0.031) remained the only significant cofactors (Table 3).

**Table 2:** Comparison of clinical parameters between patients undergoing surgery within multimodality treatment (MMT) or other treatment approaches

	MMT (*n* = 76)		Other approaches (*n* = 134)		*p*
	*n*	%	*n*	%	
Gender (male)	57	75	110	82.1	NS
Mean age	60 years (±11.3)		71 years (±9.1)		0.001
Mean Karnofsky index	87.7		83.2		0.017
Asbestos exposure	39	51.3	70	52.2	–
Histology					
Epithelioid Biphasic Sarcomatoid Not specified	62 8 3 3	81.6 10.6 3.9 3.9	79 20 13 22	59 14.9 9.7 16.4	0.001
IMIG Staging					
I II III IV	12 15 17 11	15.8 19.7 22.4 14.5	14 8 23 50	10.4 6 17.2 37.3	0.001

*NS* not significant, *IMIG* International Mesothelioma Interest Group, *MMT* multimodality treatment

**Table 3:** Multivariate Cox regression analysis for survival adjusted for clinical factors

		HR	*p*	95 % CI	
				Lower	Upper
Gender	Female	1	–	–	–
	Male	0.57	0.074	0.31	1.06
Age	>65	1	–	–	–
	<65	1.61	0.10	0.91	2.86
Karnofsky index (continuous)		0.96	**0.001**	0.94	0.96
Histology	Epithelioid	1	–	–	–
	Non-epithelioid	1.80	**0.031**	1.06	3.07
Stage	Early	1	–	–	–
	Late	1.36	0.27	0.79	2.35
Treatment	MMT	1	–	–	–
	Other	1.72	0.075	0.95	3.12

*MMT* multimodality treatment, *HR* hazard ratio, *CI* confidence interval

## Discussion

The diagnosis, staging, and treatment of MPM patients still remain a challenging task for all involved physicians. Guidelines and recommendations can help to facilitate clinical practice. In order to establish a basis for developing such clinical guidelines, meticulous and detailed MPM patient data collection is mandatory. Therefore, we established the AMIG database in 2011 aiming to improve MPM patient care and management in Austria. Furthermore, the analysis of the data should provide a general overview of the current situation and patterns of care for MPM patients all over the country. In the present report on the prospective MPM database of the AMIG, we were able to analyze a large cohort of 210 consecutive patients treated in several Austrian hospitals.

Although the usage of asbestos is forbidden in many countries worldwide, previous exposure to asbestos remains the main risk factor for the development of MPM. In our cohort, asbestos exposure was confirmed in 69 % of the included patients. In almost all cases, patients had occupation-related asbestos exposure and these were predominantly male patients. This is well in line with the published literature with a series from France, a recent paper on a large cohort from Belgium, The Netherlands, and England, and a series from Australia on MPM epidemiology [7–9]. This is particularly important since the development of MPM has a latency period of up to 30 years from the time of exposure and the peak of MPM incidence is expected to be between 2020 and 2025 in Europe. For this reason, early diagnosis and treatment will become even more important in the future, especially in patients with previous occupational exposure. To date, there are no validated biomarkers for early MPM detection available and hence appropriate early diagnostic and staging procedures are mandatory in patients suspected of having MPM. With regard to diagnosis, 78 % of patients in the present series were diagnosed via pleural biopsy. Video-assisted thoracoscopic pleural biopsy was the procedure of choice for obtaining histological verification in the majority of cases as recommended by the latest published guidelines [4, 10, 11]. The epithelioid subtype was the most common histology, as in all larger published series and in the database of the International Association for the Study of Lung Cancer (IASLC) [8, 12–14].

Appropriate staging of MPM can be challenging and is important for therapeutic decision making. Staging by PET/CT was undertaken in 64 % of patients, representing the most common staging procedure for this disease in Austria. According to the literature, it is mainly recommended as part of a study protocol in order to locate tumor sites, distant metastasis, and possible nodal involvement, and is useful for assessing early response to treatment. As advocated by many different clinical guidelines, the most recent TNM-based IMIG classification was commonly used in this cohort [15]. Further staging methods such as magnet resonance imaging (MRI), exploratory laparoscopy, or mediastinoscopy/endobronchial ultrasound fine needle aspiration (EBUS-TBNA) have not been applied uniformly. As there is no clear evidence of these methods in the staging of MPM, this remains an area for further investigation.

The majority of the patients in the AMIG cohort were diagnosed with MPM of stages III and IV, and therefore chemotherapy was the most common treatment option in this series. In all, 100 patients received the combination of platinum and pemetrexed, which has been the standard chemotherapy protocol used since 2003 worldwide [16]. However, other chemotherapy protocols were also used, according to the physician’s choice. Symptom control by best supportive care was achieved in 30 patients. This is particularly important since older patients with reduced performance status are not able to undergo therapy with curative intention and hence improvement of quality of life becomes even more important.

The impact of surgery on patient survival in MPM has been well described in the past and is a particularly important component in multimodality treatment protocols [12, 13, 17]. Macroscopic complete resection (MCR) has been defined as the ultimate goal for surgery with curative intent [18]. In the AMIG cohort, 76 patients received MCR as part of a multimodality protocol. With regard to surgical procedures, extrapleural pneumonectomy (EPP) was the most common surgical technique followed by pleurectomy/decortication (P/D). Both techniques have been standardized in the framework of a consensus report on surgical techniques [15]. Postoperative complications were present in 21.8 % of patients and 30-day mortality was nil in this analysis. These data are compare well with large published series on the outcome of different surgical techniques [13, 19–22].

Despite tremendous efforts in all fields of therapy, especially surgery and systematic treatment, the survival of MPM patients still remains poor. Only well-selected patients can achieve more than 3 years of survival. In our unselected patient cohort, the median OS was 19.1 months with a 3- and 5‑year survival of 30 % and 23 %, respectively. These survival rates are in line with or superior to the survival data of larger prospective and retrospective studies in MPM treatment [5, 8, 9]. Analysis of prognostic factors for survival in the AMIG database revealed that the Karnofsky index at the time of diagnosis, early stages according to the IMIG staging system, epithelioid histology, and surgery within multimodality treatment had a favorable impact on outcome. Similar co-factors have been determined by the IASLC database and are part of two widely used prognostic scores [14, 23]. Interestingly, there was no difference in survival in patients according to age. Moreover, we did not observe any significant survival difference with regard to gender. However, in a recent publication, a threefold better survival rate in female than in male patients was reported, irrespectively of age, stage, and treatment [24].

In summary, we report on the first nationwide mesothelioma database in Austria. Our initial analysis reveals comparable practice in the management of MPM patients in different participating institutions. The diagnosis, staging, treatment, and outcome of this patient cohort are well in line with international guidelines and experiences of larger centers worldwide. However, these data serve as a starting point for future collaborative efforts within Austria. Prospective clinical studies, especially on new treatment strategies, are warranted to optimize outcome in this devastating disease.
